# Thymoquinone Induces Telomere Shortening, DNA Damage and Apoptosis in Human Glioblastoma Cells

**DOI:** 10.1371/journal.pone.0012124

**Published:** 2010-08-12

**Authors:** Resham Lal Gurung, Shi Ni Lim, Aik Kia Khaw, Jasmine Fen Fen Soon, Kirthan Shenoy, Safiyya Mohamed Ali, Manikandan Jayapal, Swaminathan Sethu, Rajamanickam Baskar, M. Prakash Hande

**Affiliations:** 1 Department of Physiology, Yong Loo Lin School of Medicine, National University of Singapore, Singapore, Singapore; 2 Division of Cellular and Molecular Research, Department of Radiation Oncology, National Cancer Centre, Singapore, Singapore; University of Medicine and Dentistry of New Jersey, United States of America

## Abstract

**Background:**

A major concern of cancer chemotherapy is the side effects caused by the non-specific targeting of both normal and cancerous cells by therapeutic drugs. Much emphasis has been placed on discovering new compounds that target tumour cells more efficiently and selectively with minimal toxic effects on normal cells.

**Methodology/Principal Findings:**

The cytotoxic effect of thymoquinone, a component derived from the plant *Nigella sativa*, was tested on human glioblastoma and normal cells. Our findings demonstrated that glioblastoma cells were more sensitive to thymoquinone-induced antiproliferative effects. Thymoquinone induced DNA damage, cell cycle arrest and apoptosis in the glioblastoma cells. It was also observed that thymoquinone facilitated telomere attrition by inhibiting the activity of telomerase. In addition to these, we investigated the role of DNA-PKcs on thymoquinone mediated changes in telomere length. Telomeres in glioblastoma cells with DNA-PKcs were more sensitive to thymoquinone mediated effects as compared to those cells deficient in DNA-PKcs.

**Conclusions/Significance:**

Our results indicate that thymoquinone induces DNA damage, telomere attrition by inhibiting telomerase and cell death in glioblastoma cells. Telomere shortening was found to be dependent on the status of DNA-PKcs. Collectively, these data suggest that thymoquinone could be useful as a potential chemotherapeutic agent in the management for brain tumours.

## Introduction

A major concern of cancer chemotherapy is the side effects induced by the non-specific targeting of both normal and cancerous cells by chemotherapeutic drugs. Much emphasis has been placed on discovering new compounds that specifically target cancer cells with minimal toxicity to normal cells. Recently various natural compounds have been shown to be promising chemotherapeutic agents with lesser cytotoxicity to the normal cells [1]. Thymoquinone (TQ) is the main active constituent found in the crude extracts of the seeds of *Nigella sativa Linn.* and studies have shown that TQ possesses antineoplastic properties [2], [3], [4]. Decrease in cell survival with pro-apoptotic properties have also been identified in various cancer cell lines including the canine osteosarcoma cells, human colon carcinoma, breast adenocarcinoma cells, and the ovarian adenocarcinoma cells [5]. TQ has been demonstrated as a cytotoxic agent in several multi-drug resistant human tumour cell lines [6]. However, the underlying molecular mechanism of its anticancer properties is not well understood.

Telomerase activity is generally absent in human somatic cells whereas in 90% of tumour cells, activation of telomerase prevents telomere shortening and thus allows for unlimited replicative capability [7]. Telomerase inhibition therefore presents an attractive target for cancer therapeutics [8], [9]. Early events in the development of glioblastoma include the re-activation of human telomerase reverse transcriptase (hTERT) [10]. In addition, a p53 mutation also contributes to the progression to malignancy in gliomas [11]. Studies have shown that human glioblastoma cells deficient in DNA-PK activity are able to recruit a slow, error prone repair process that can result in the increased formation of chromosome aberrations [12]. DNA-dependent protein kinase is a nuclear, serine/threonine protein kinase consisting of a 470-kDa catalytic subunit (DNA-PKcs) and a heterodimeric regulatory complex ku70/80.This enzyme is essential for the repair of DNA double-strand breaks and it mediates repair via phosphorylation of downstream DNA binding proteins such as p53, DNA ligase IV and transcription factors such as Fos, Jun, myc, Oct1, NF-kappa B, and RNA polymerase H [13], [14].

Brain tumours have low prognosis with survival rate of only few months and traditional therapy methods such as surgery, radiotherapy and chemotherapy have low efficacy. Furthermore, brain tumour cells are resistant to numerous chemotherapeutic agents. Inhibition of DNA repair proteins has been suggested to increase the sensitivity of DNA damaging chemotherapeutic agents. In this study, we sought to determine the anti-cancer effects of TQ in a defined DNA repair proficient and DNA repair deficient cells and its impact on the telomerase-telomere status in DNA-PKcs proficient and deficient human brain cancer cells. We performed *in vitro* studies to determine the level of cytotoxicity induced by TQ in human glioblastoma cells. In addition, the effects of TQ on both the telomerase activity and telomere length in cancer cells with different DNA-PK status were also investigated. Our findings demonstrate that at selective dose of TQ, glioblastoma cells were more sensitive to TQ-induced damage as compared to normal cells as indicated by the higher levels of apoptosis and reduced cell viability. Increased expression of pro-apoptotic proteins Bax and cytochrome *c* were also observed in glioblastoma cells. In addition, DNA-PKcs proficient glioblastoma cells were more sensitive to TQ as compared to DNA-PKcs deficient glioblastoma cells.

## Materials and Methods

### Cells, Cell Culture and Drug Treatment

Two human glioblastoma cell lines, M059K (CRL-2365) and M059J (CRL-2366) (American Type Culture Collection, USA) were used in the study. M059J cells lack DNA-PKcs activity while M059K cells express normal levels of DNA-PKcs. Glioblastoma cells were cultured in Dulbecco's Modified Eagles Medium (DMEM) supplemented with 10% heat inactivated foetal bovine serum (Hyclone, USA) and 100 U/ml of penicillin/streptomycin (Gibco, USA). hTERT immortalised human foreskin fibroblasts (hTERT-BJ1; Clontech, USA) were cultured in 4∶1 ratio of DMEM and Medium 199, supplemented with 10% foetal bovine serum, 100 U/ml of penicillin/streptomycin, 1% sodium pyruvate and 2% L-glutamine (Gibco, USA). Normal human lung fibroblasts (IMR-90; Coriell Cell Repositories, USA) were cultured in Minimal Essential Medium (Gibco, Invitrogen, USA) supplemented with 15% foetal bovine serum, 100 U/ml of penicillin/streptomycin, 1% vitamins and 1% essential and non-essential amino acids. All cells were maintained in a humidified 5% CO_2_ incubator at 37°C. Stock solution of thymoquinone (TQ) (Sigma, USA) and a DNA-PKcs inhibitor NU7026 (Calbiochem, USA) [15] were prepared in dimethyl sulfoxide (DMSO) and suitable working concentrations were made from the stock using complete medium. Exponentially growing cells were treated with 0 to 200 µM TQ for 24 hours following which 25 and 50 µM doses were selected for subsequent studies.

### Assay for cell viability

Following TQ treatment, attached cells were washed once with phosphate buffered saline (PBS). Crystal violet solution (0.75% crystal violet in 50% ethanol: distilled water with 1.75% formaldehyde and 0.25% NaCl), which stains DNA by binding electrostatically to nuclear proteins was added to the wells and incubated for 20 minutes at room temperature. Following PBS washes, to remove excess crystal violet solution, wells were air dried. Sodium dodecyl sulphate: PBS solution was added to lyse the cells and solubilise the dye. We measured the amount of crystal violet taken up by cells at 595 nm absorbance using an automated ELISA reader.

### Cell cycle analysis

Following TQ treatment, cells were harvested, washed in 0.1% BSA: PBS, fixed in 70% ethanol: 1 × PBS, and stained with propidium iodide (Sigma, USA): RNase A (Roche, USA) (2 mg propidium iodide and 2 mg RNaseA/100 mL 0.1% BSA in 1 × PBS). Samples were analysed by flow cytometry (FACSCalibur™, Becton Dickinson, USA) at 488 nm excitation *λ* and 610 nm emission *λ*. A total of 10,000 events were captured. Data obtained was analysed using WINMDI software.

### Analysis for apoptosis and necrosis

Untreated and TQ-treated glioblastoma cells were stained with an Annexin V antibody and propidium iodide using Annexin-V-FITC staining kit (Sigma, USA). Samples were then analysed by flow cytometry. A total of 10,000 events per sample was obtained and the data was analysed using WINMDI software.

### Protein estimation by Western blot analysis

Total cellular proteins were isolated using RIPA (radio-immunoprecipitation assay) buffer (1% nonidet P-40, 1% sodium deoxycholate, 0.1% SDS, 0.15 M NaCl, 0.01 M sodium phosphate, 2 mM EDTA, 50 mM sodium fluoride, 0.2 mM sodium vanadate and 100 U/ml aprotinin, pH 7.2) from control and treated cells. The whole cell lysate was recovered by centrifugation at 14,000 rpm for 10 minutes. Protein concentration was determined by the bicinchoninic acid method using an assay kit (Pierce Biotechnology, USA) with bovine serum albumin as a standard. Western blot analyses of cell cycle regulatory proteins (p53, p21), pro-apoptotic factors (Bax, Cytochrome *c*), hTERT and β-actin (Santa Cruz Biotechnology, USA) were performed using specific antibodies.

### Assessment of DNA damage

TQ induced DNA damage was evaluated using alkaline single cell gel electrophoresis (Comet) assay. Following treatment with TQ for 24 hours, the extent of total DNA damage was evaluated as described previously [16]. Cells were harvested and resuspended in Hank's Balanced Salt Solution (Sigma, USA) with 10% DMSO and 0.5 M EDTA. The cell suspension was then suspended in 0.7% low melting agarose at 37°C (Conda, Spain), and layered on to comet slides (Trevigen, USA). The cells were then lysed in lysis solution containing 2.5 M NaCl, 100 mM pH 8.0 EDTA, 10 mM Tris-HCL, 1% Triton –X at 4°C for 1 hour. Denaturation was carried out for 40 minutes, in chilled alkaline electrophoresis buffer (pH 13.0–13.7). Electrophoresis was subsequently carried out for 20 minutes. Slides were immersed in neutralization buffer (500 Mm Tris-HCL, pH 7.4), dehydrated, dried and stained with SYBR Green dye (Trevigen) and scored with Comet Analysis Software (Metasystems, Germany). The images were captured using Zeiss Axioplan 2 imaging fluorescence microscope (Carl Zeiss, Germany) equipped with triple band filter. Fifty comets per sample were randomly selected and analysed. The extent of DNA damage was expressed as tail moment, which corresponded to the fraction of the DNA in the tail of the comet.

### Measurement of Telomerase Activity

Telomerase activity was assessed using Telomeric Repeat Amplification Protocol (TRAP) using TRAPeze® XL Telomerase Detection Kit (Chemicon International, USA). All steps were done according to the manufacturer's instructions with some modifications. Briefly, total protein was extracted using CHAPS lysis buffer provided and 1.5 µg protein was treated with 1 µl/ml RNase inhibitor to perform PCR reaction. The PCR was initiated by using the telomerase mediated elongation products (i.e. the telomeric DNA repeats) as template. Subsequently normal PCR cycle was performed using primer pairs with quenched fluorescein to amplify the telomeric DNA repeats. Fluorescence signals were generated by unquenching the fluorescein on PCR primers, and the fluorescence signals were measured by fluorescence multi-well plate reader. Fluorescence signals of PCR products were measured using fluorescence plate reader TECAN SpectraFluor Plus.

### Telomere length analysis

#### Telomere Restriction Fragment (TRF) Length Analysis

DNA extraction was performed according to the manufacturers' protocol using DNeasy Tissue Kit (Qiagen, USA). The telomere restriction fragment length analysis assay was performed using Telo-TAGGG Length Assay Kit (Roche Applied Science, USA). Two micrograms of pure genomic DNA was digested with *Hinf1* and *Rsa1* restriction enzymes for 2 hours at 37°C. These restriction enzymes digest the whole genomic DNA except the sub-telomeric and telomeric regions. Digested DNA fragments were separated by gel electrophoresis in 0.8% agarose gel at 60 V for 3 hours, transferred via overnight Southern blot onto a nylon membrane and cross-linked onto the membrane using a UV cross-linker (Stratagene, USA). Telomere restriction fragments were hybridised with telomere-specific digoxigenin-labelled probe and incubated with Anti-DIG alkaline phosphatase and tetramethylbenzidine according to manufacturer's protocol. The chemiluminescence signals were scanned by the Kodak Gel imaging system and analyzed by the Kodak imaging software to calculate the quantitative measurements of the mean TRF length.

#### Quantitative Fluorescence *in situ* Hybridisation (qFISH) Analysis

Cells were arrested at mitosis by treatment with colcemid (0.1 µg/ml). Cells were subsequently incubated with a hypotonic solution of potassium chloride at 37°C for 15 minutes followed by fixation in Carnoy's fixative. Quantitative fluorescence *in situ* hybridisation (qFISH) was performed using telomere sequence-specific peptide nucleic acid (PNA) probe labelled with Cy3 as described [17], [18]. Metaphase spreads for different samples were hybridised simultaneously. To avoid selection bias, good and well spread metaphases were randomly chosen for analysis. Images were acquired on the same day (within four hours) for all the samples using the Zeiss Axioplan 2 imaging fluorescence microscope. Fluorescence intensity of telomere signals was measured in 10–15 metaphases using the *in situ* imaging software (Metasystems, Germany).

### Gene expression analysis

The total RNA was extracted from M059K and M059J cells using QIAmp RNA Blood Mini Kit (Qiagen, Hilden, Germany). The extracted RNA was quantified using NanoDrop 1000 (Thermo Scientific, USA). RNA integrity was checked using Bio-Analyzer (Agilent Technologies, Inc., USA). Five hundred nanograms of extracted RNA from each sample were used for gene expression study. TotalPrep RNA Amplification Kit (Ambion Inc., TX, USA) was used for cRNA amplification process. The biotinylated amplified RNA thus generated was used for hybridization with HumanRef8 V3.0, Human Whole-Genome Expression BeadChips (Illumina Inc., USA) for 16 hours at 58°C. After the incubation period, the arrays were washed and stained with Streptavidin-Cy3 (GE Healthcare, Bio-Sciences, UK). Illumina Bead Array Reader was used to scan the arrays. The array data thus obtained after scanning was imported and analysed using Partek® Genomics Suite™ (Partek GS) (Partek Incorporated, MO, USA).

### Chromosome analysis by multicolour fluorescence *in situ* hybridisation (mFISH)

The experimental procedure is outlined in our earlier publications [19], [20]. Chromosome paints were obtained from MetaSystems GmbH, Germany. After pepsin treatment (1 g/50 ml; Sigma) for about 2 minutes at 37°C the chromosomes were post-fixed in 1% formaldehyde in 1XPBS with 50 mM MgCl_2_ for 10 minutes at room temperature. The chromosomes are stabilised in SSC prior to the denaturation and were subsequently denatured with a basal solution (0.07 N NaOH). Afterwards, the chromosomes were rinsed in SSC buffer again to stop the denaturation process and to stabilize their structure. The probe cocktail was denatured 75°C for 5 minutes. The probe was allowed to pre-hybridise for half an hour at 37°C to reduce unspecific binding of short or repetitive DNA pieces. The denatured and prehybridised probe cocktail was applied onto the denatured chromosome preparation, overlaid with a coverslip and sealed with rubber cement. The slides were then incubated at 37°C in a humidified chamber for 72 hours. Post-hybridisation washing was done in 1xSSC at 75°C for 5 minutes after removing the coverslips. Slides were incubated in 4xSSCT for 5 minutes and blocked with blocking reagent at 37°C for 10 minutes. Subsequently, slides were incubated with detection 1+3 reagent to detect Cy5 which is indirectly labelled. Slides were then counterstained with DAPI (Metasystems). Microscopic analysis was performed using an Axioplan II imaging microscope (Carl Zeiss) with an HBO-103 mercury lamp and filter sets for FITC, Cy3.5, Texas Red, Cy5, Aqua, and DAPI. Images were captured, processed, and analyzed using ISIS mFISH imaging software (MetaSystems). In the mFISH technique, all 23 chromosomes (1–22 and X) are each painted in a different colour, using combinatorial labelling, so that any interchromosomal translocations are observed as colour junctions on individual chromosomes.

### Statistical Analysis

Statistical significance in the data sets was assessed by Student's *t*-test using Microsoft Excel 2003 (Microsoft Corporation, USA) and two-way ANOVA using Graphpad Prism. The difference was considered to be statistically significant when p<0.05.

## Results

### Thymoquinone reduced the viability of the human brain cancer cells more effectively than non-cancerous cells

**Figure 1:**
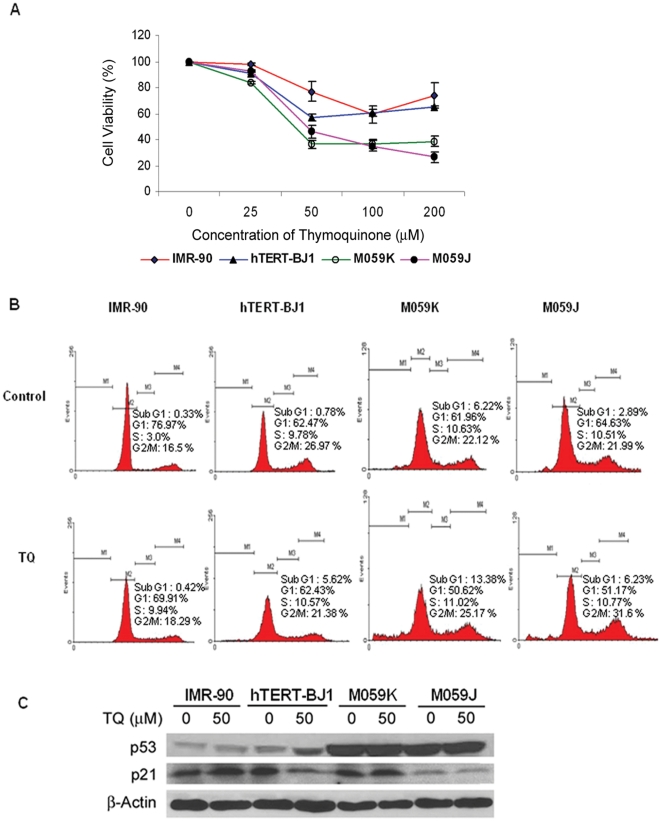
Thymoquinone induces cell death in human glioblastoma cells. (**A**) Cell viability of the various cell types after 24 hour treatment with TQ at the different concentrations is shown. The percentage cell viability was normalised against the DMSO controls (0 µM) for each cell type. Mean and standard deviations of the three independent experiments are shown. (**B**) Cell cycle profiles of the IMR 90, hTERT-BJ1 and glioblastoma cells without (0 µM) or with TQ (50 µM) treatment as measured by propidium iodide staining. (**C**) Changes in level of cell cycle regulatory proteins in the cell lines tested. Whole cells were lysed and equal amounts of proteins were separated using 4–20% SDS-PAGE, transferred to PVDF membrane and immunoreacted with antibodies against p53 and p21. β-actin was used as loading control.

Exposure to TQ resulted in a dose dependent decrease in cell survival in all the cell lines from 0 to 50 µM of TQ (Figure 1A). The glioblastoma cells displayed greater TQ induced cell death compared to the normal cells tested. The greatest difference in survival between the normal cells and glioblastoma cells following TQ treatment occurred at concentration of 50 µM. The death induced in glioblastoma cells was significantly higher than normal (IMR90) cells (Figure 1A). Based on the viability trend obtained, 50 µM was chosen for subsequent assays. Furthermore at 50 µM, DNA-PKcs proficient M059K cells were more sensitive TQ-induced cell death as compared to DNA-PKcs deficient (M059J) cells.

To determine whether the growth inhibition by TQ involves cell death or growth arrest, cells stained with PI were subjected to FACS analysis. Increase in sub-G1 population, an indication of apoptotic death, was observed only in telomerase positive cells (Figure 1B). At 50 µM TQ, the glioblastoma cells exhibited higher percentage of apoptotic cell death (sub-G1 population) than the normal IMR90 cells (Figure 1B). There was a two fold increase in glioblastoma cell death following TQ treatment compared to untreated controls. In addition, M059J cells exhibited G2/M arrest (about 10%). In contrast, a three-fold increase in S-phase population in normal cells treated with TQ was observed. However, in telomerase positive (hTERT-BJ1) cells, there was a 5 fold increase sub-G1 population following TQ treatment. Impairment of growth in normal cells following TQ treatment was accompanied by the increase in p53 and p21 proteins (Figure 1C). However, hTERT-BJ1 cells showed an increase in p53 protein level followed by a decrease in the expression of cyclin dependent kinase inhibitor protein, p21.

### Thymoquinone induces apoptotic death in glioblastoma cells

**Figure 2:**
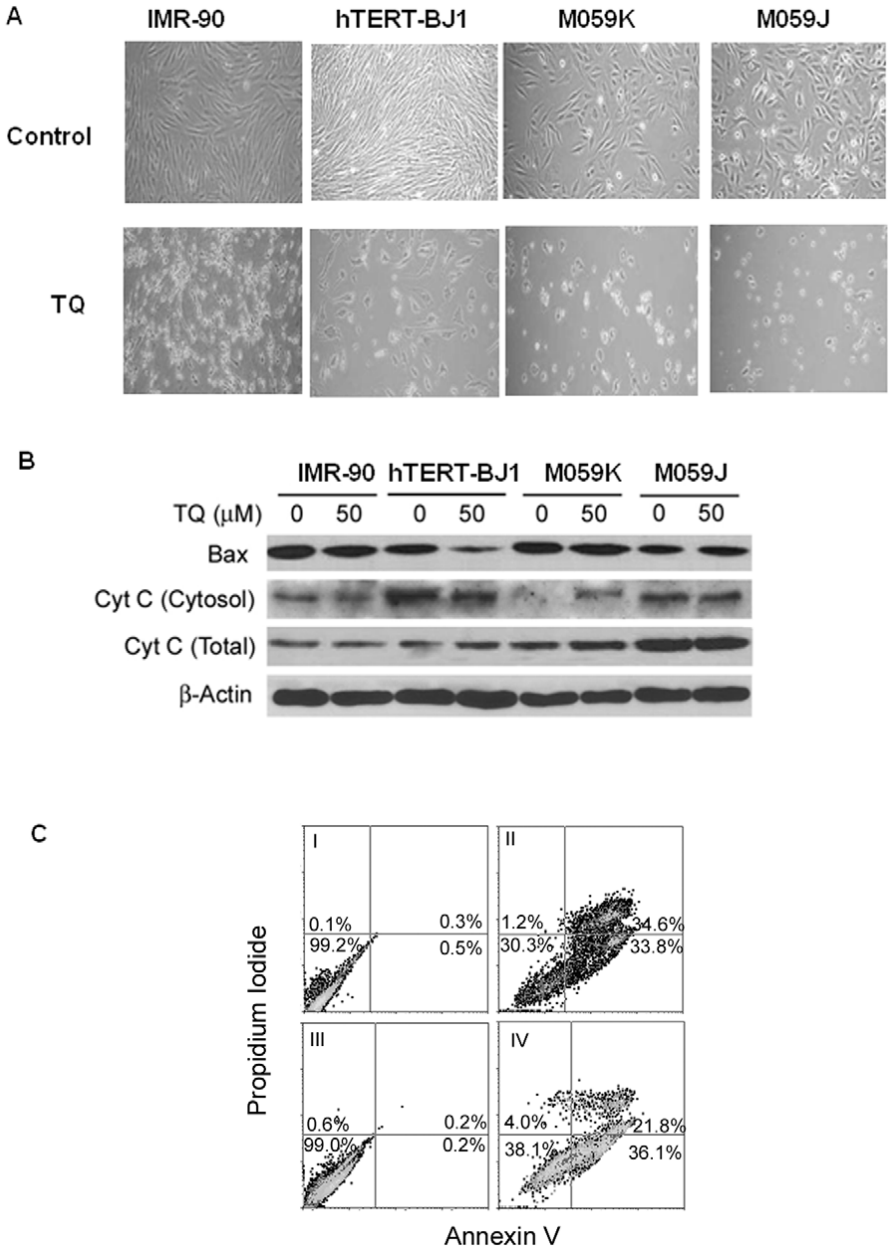
Thymoquinone treatment produces apoptosis in human glioblastoma cells. (**A**) Morphological appearance of cells treated with TQ (50 µM) for 24 hours. (**B**) Changes in the level pro-apoptotic proteins Bax and cytochrome *c* in different cell types used in the study. (**C**) FACS profiles of staining for Annexin V and propidium iodide to determine apoptosis and necrosis. (**l**) M059K (untreated) (**ll**) M059K (TQ treated) (**lll**) M059J (untreated) and (**IV**) M059J (TQ treated).

As shown in Figure 2A, all tested cells treated with 50 µM TQ exhibited change in morphology compared to their respective controls. However, more floating and rounded cells which suggest dying cells were observed in glioblastoma cells. Expression of pro-apoptotic Bax protein level increased only in glioblastoma cells (M059K and M059J) following 50 µM TQ (Figure 2B). To investigate the apoptotic cascades involved in the TQ-induced cell death in glioblastoma cells, total and cytosolic cytochrome *c* protein level was measured. The level of cytosolic cytochrome *c*, which is critical for the initiation of apoptosis, was significantly increased in M059K cells (Figure 2B). Cell death following TQ treatment in glioblastoma cells was predominantly due to apoptosis as demonstrated by the increase in cell population showing positive staining for Annexin V (Figure 2C).

### Thymoquinone produces significant DNA damage in a dose dependent manner

**Figure 3:**
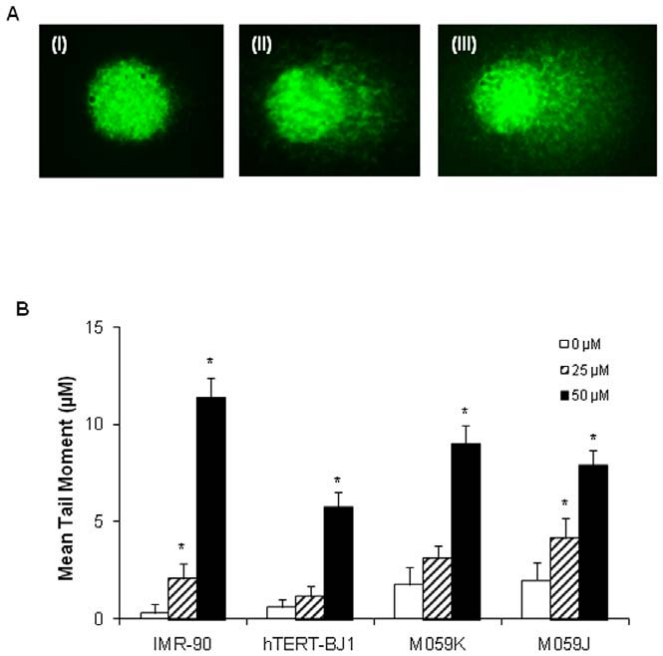
Induction of DNA damage in thymoquinone treated glioblastoma cells. DNA damage as measured by the comet assay in different cell types following treatment with thymoquinone at 25 µM and 50 µM for 24 hours. The damage distribution was measured as tail moment (product of tail length and fraction of DNA). (**A**) Representative of SYBR Green-stained comets prepared from control (**I**) and TQ treated glioblastoma cells (**II** and **III**). Varying degree of damage could be seen (**II** and **III**). (**B**) Extent of damage is presented by mean tail moment (product of tail length and fraction of DNA) in the attached cells. (*) indicates that the change in DNA damage with respect to control is statistically significant, i.e. p-value <0.05. The increase in DNA damage between 25 µM and 50 µM was also found to be statistically significant for all samples. Tail moment (in microns) is given. Mean and standard error from three independent experiments are shown.

Single-cell gel electrophoresis (known as comet assay) under alkaline condition (pH>13) was used to determine the DNA damage induced by TQ (Figures 3A and B). Different types of DNA damage such as double-strand breaks, single-strand breaks and alkali labile sites were measured using comet assay. Representative images of comet analysis were shown in Figure 3A. Following TQ treatment, all cells showed a dose dependent increase in DNA damage compared to respective controls (Figure 3B). At 50 µM TQ, M059J cells were less susceptible to TQ induced DNA damage compared to M059K cells. Interestingly, DNA damage was lower at 25 µM TQ in normal fibroblast cells (IMR90 and hTERT-BJ1) than in glioblastoma cells. On the contrary, TQ treated M059J cells showed significant DNA damage unlike TQ treated M059K cells that displayed minimal increase when compared to respective untreated control cells at 25 µM TQ (Figure 3B). The DNA damage data obtained by comet assay were supported by higher micronuclei detected in the TQ treated cells (data not shown) which are suggestive of both DNA damage and resulting chromosome instability.

### Thymoquinone induces telomere attrition

**Figure 4:**
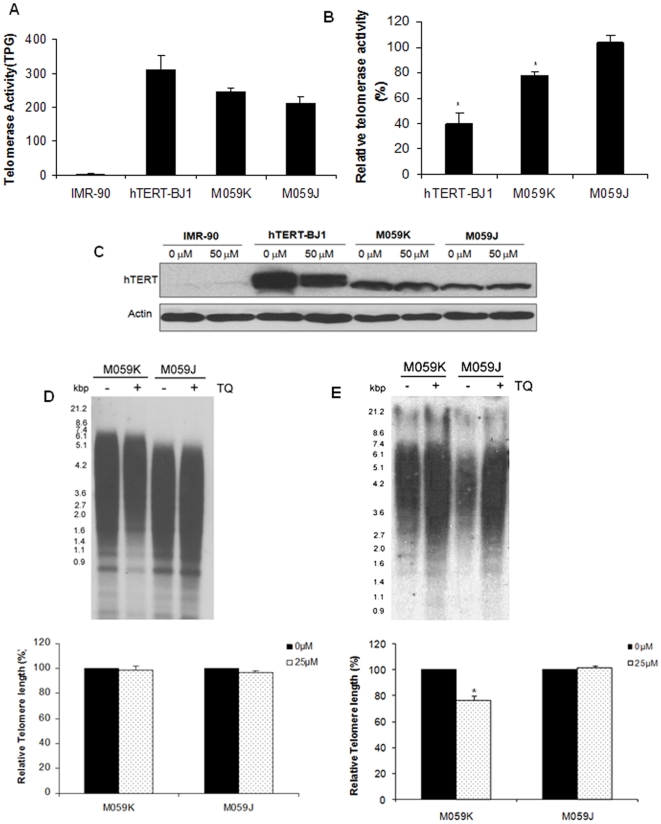
Thymoquinone treatment causes telomere attrition in glioblastoma cells. (**A**) Basal telomerase activity level in IMR90, hTERT-BJ1, M059K and M059J cells. (B) Percentage change in telomerase activity relative to their respective untreated controls following exposure to 50 µM TQ for 24 hours. (**C**) Expression of hTERT following TQ treatment (50 µM) for 24 hours. Telomere restriction fragment analysis for telomere length measurement in glioblastoma cells following 4 days (D) and 15 days (**E**) treatment with 25 µM TQ. Changes in telomere length are expressed as percentage with respect to its controls. Graph shows mean and standard error from three independent experiments and TRF blot shown is a representative from three independent experiments. (_*_) indicates statistical significance, p<0.05.

Our findings from telomerase positive hTERT-BJ1 fibroblasts demonstrate increased sensitivity to TQ induced anti-proliferative effect as compared to normal cells. To determine whether the decrease in cell proliferation was linked to effect of TQ on telomerase activity, we evaluated the level of telomerase activity in cells with and without TQ treatment. Firstly, the basal level of telomerase activity in the different cells was investigated. As expected, telomerase activity was negligible in normal lung fibroblasts (IMR90), while the hTERT-BJ1 and glioblastoma cells showed positive telomerase activity (Figure 4A). M059J cells displayed lower telomerase activity compared to M059K cells. At 24 hours post-TQ treatment (50 µM), a significant decrease in telomerase activity was detected in hTERT-BJ1 and M059K but not in M059J cells (Figure 4B). Furthermore, we have observed that there is a decrease in the expression of hTERT following TQ treatment (Figure 4C) demonstrating a novel effect of TQ on telomere-telomerase complex.

Since, telomerase is pivotal for the maintenance of telomere length, we monitored changes in telomere length, if any, following TQ treatment using TRF assay. Lower dose (25 µM) of TQ was used for an extended time of 15 days in glioblastoma cells and telomere length was measured at 4 and 15 days of treatment. Only glioblastoma cells were evaluated for telomere length analysis because our findings have shown that the level of TQ- induced DNA damage (Figure 3) and anti-proliferative effect (Figure 1) of TQ were more pronounced in glioblastoma cells as compared to normal cells. Short term treatment for 4 days with 25 µM TQ did not show detectable change in telomere length (Figure 4D). Following TQ (25 µM) treatment for 15 days, M059K cells showed greater telomere shortening compared to M059J cells (Figure 4E). Thus the data suggest that TQ disrupts telomere length maintenance by inhibiting the activity of telomerase over time in cancer cells. However, M059J cells deficient in DNA-PKcs were less sensitive to TQ mediated effects on telomere length maintenance. Therefore, we investigated the role of DNA-PKcs in TQ mediated effects on telomeres.

### Role of DNA-PKcs in thymoquinone mediated telomere attrition

In order to understand the influence of DNA-PKcs on the TQ mediated effects on telomeres in the glioblastoma cells, a two pronged approach was taken. Firstly, we profiled M059K and M059J cells for inherent similarities and differences by gene expression profiling and karyotyping. Secondly, we validated the role of DNA-PKcs by the using its specific pharmacological inhibitor, NU7026 [15].

**Figure 5:**
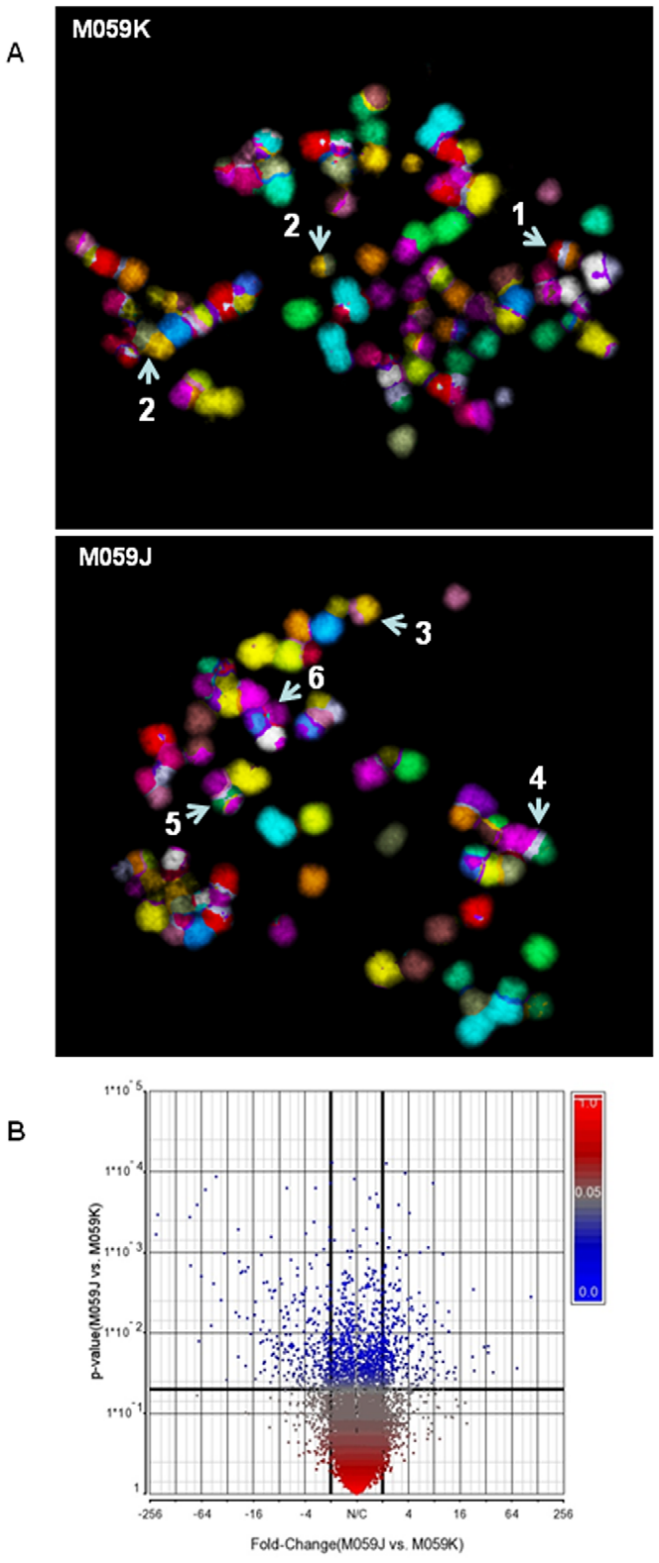
Chromosome rearrangements and gene expression profiles in M059K and M059J cells. (**A**) Cells were arrested at metaphase and chromosome spreads were subjected to multicolour fluorescence *in situ* hybridisation (mFISH). Representative mFISH karyotypes of M059K and M059J cells. Note the higher number of chromosome re-arrangements in both the cell types. Recurrent chromosome translocations are numbered as follows: (1) - t(8;21;5); (2) - t(16∶10); (3)- t(16; 17); (4) - t(9;21;3); (5) – t(9;21;19); (6) – t(14;Y). ((**B**) Differential gene expression profiles in M059K and M059J cells. Volcano plot indicates differentially regulated genes. Clusters in blue indicate genes differentially genes (p<0.05; 1 way ANOVA). Black bold lines (X axis) indicate fold change cut off of 2.

It is reported that M059J cells were derived from M059K cells and were deficient in DNA-PKcs [21], [22]. A detailed characterisation and gene expression profiles of these cells have not been reported so far. mFISH was performed to karyotype M059K and M059J cells. Modal chromosome number for M059K cell type is to be 75 (with a range of 65 to 79 (www.atcc.org) with a polyploidy rate of 22% whereas M059J cells reported to be aneuploid (www.atcc.org). Representative mFISH images of M059K and M059J metaphase spreads are shown in Figure 5A. Our analysis has revealed that average chromosome number for M059K is 87 and that of M059J is 68. The karyotypes clearly indicate complex chromosomal rearrangements like reciprocal and non-reciprocal translocations in both the cell types. Recurrent chromosomal translocations such as t(8;21;5), t(1;21;6), t(5;21) and t(4;17;22;21;9) were detected in M059K cells. Likewise, translocations such as t(7;16;14); t(11;12); t(8;6); 2 copies of t(9;21;3), and two copies of t(9;21;19) were detected in M059J cells. Translocations such as t(16;17), t(16;10) and multiples copies of t(9;21) were commonly seen in both the cell types. At this moment, it is not possible for us to conclude that the translocations detected in M059J cells are result of defective DNA-PKcs. Following microarray analysis to determine the gene expression profiles of M059K and M059J cells, we found that a total of 587 genes (approximately 2% of the genes analysed) were differentially expressed based on the set criteria (p<0.05; fold difference of 2) as indicated in Figure 5B. This might suggest that genetic differences exist, but minimal, among the two different cell types (data not shown).

**Figure 6:**
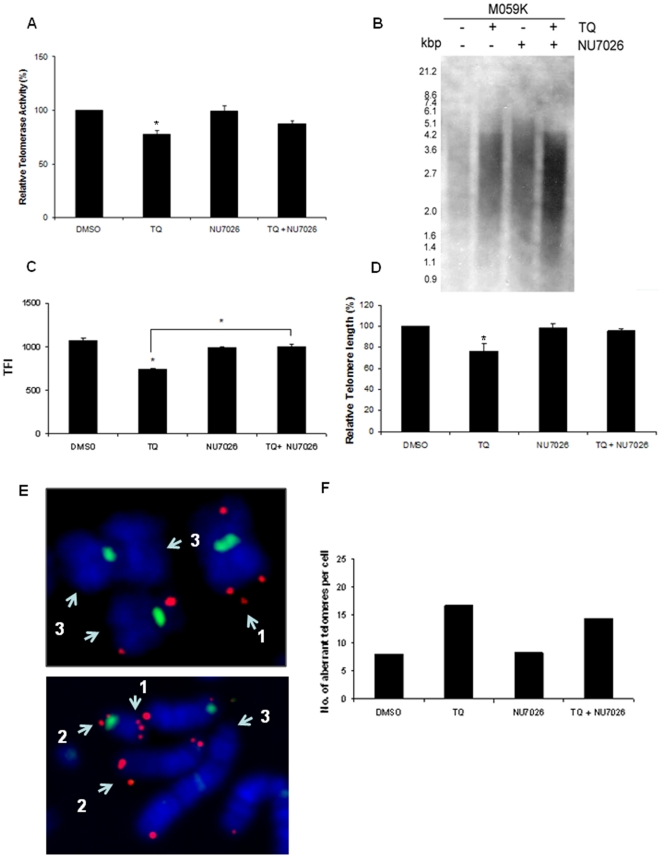
Role of DNA-PKcs in thymoquinone mediated telomere attrition. (**A**) Percentage change in telomerase activity relative to their untreated controls following exposure to 50 µM TQ for 24 hours with and without DNA-PKcs inhibitor, NU7026 (10 µM). Telomere length measurements using TRF analysis (**B and D**) and qFISH (**C**) in glioblastoma cells following 15 day-treatment with 25 µM TQ and NU7026 (10 µM). Changes in telomere length by TRF are expressed as percentage with respect to its controls (**D**). Mean ± standard error from two independent experiments are shown. * p<0.05. (**C**) Telomere fluorescence intensity (TFI) values from qFISH analysis are displayed for different samples. (**E–F**) Telomere dysfunction induced by 15-day treatment with TQ (25 µM) in DNA-PKcs inhibited (NU7026, 10 µM) M059K cells. (**E**) Partial metaphase spreads from M059K cells displaying chromosomes stained for telomeres (red) and centromeres (green) with telomeric doublets (1), different signal intensities on sister chromatids (2) or absence of telomere signals (3). (**F**) Number aberrant telomeres per cell in DNA-PKcs inhibited M059K cells after TQ treatment.

As shown in Figures 4B and D, cells deficient in DNA-PKcs (M059J) exhibited relatively lesser TQ-mediated telomerase inhibition and telomere attrition. To further understand the role of DNA-PKcs in the differential effect of TQ on telomere-telomerase homeostasis, we used NU7026, a DNA-PKcs specific pharmacological inhibitor along with TQ. We observed that TQ induced telomerase inhibition in M059K cells which were pre-treated with NU7026 was 10% lesser than TQ alone treatment (TQ - 23% inhibition and NU7026 + TQ - 13% inhibition) (Figure 6A). Similar trend was seen with regards to TQ mediated telomere attrition and NU7026 was found to slow this process down in M059K cells (Figures 6B–D). These results were comparable to those seen in M059J cells (Figures 4A–E) highlighting the role of DNA-PKcs in TQ mediated telomere shortening in glioblastoma cells. We hypothesised that telomere attrition resulted from TQ treatment in glioblastoma cells may produce dysfunctional telomeres. Treatment with drugs may generate aberrant telomeres [23] due to dysfunctional telomeres and replication stress. To determine whether telomerase inhibition and telomere shortening in TQ treated M059K cells has resulted in telomere dysfunction, we analysed fragile telomeres in M059K cells following TQ exposure with or without NU7026. In metaphase spreads, fragile or aberrant telomeres are characterised by the presence of undetectable telomeres at chromosome ends, telomeric doublets and different signal intensities on sister chromatids. Representative metaphase images with fragile telomeres are shown in Figure 6E. Number of aberrant telomeres per cell displayed in Figure 6F corresponded to telomere attrition and telomerase inhibition (Figures 6A–D). TQ alone has resulted in elevated number of dysfunctional telomeres, while combined treatment produced a lower number of dysfunctional telomeres compared to TQ single treatment but higher than the NU7026 alone treatment (Figure 6F).

## Discussion

There has been an increasing interest in natural plant products as potential chemotherapy in human tumours due to their lower cytotoxic effects on the normal cellular system [1]. Limited number of reports is available in the literature on the growth inhibitory and pro-apoptotic effects of TQ in tumour cells [5], [24]. Moreover, effect of TQ on telomerase activity and telomere length in cancer cells has not yet been explored. We demonstrate in this study that TQ exhibits specific cytotoxicity in cancer cells at selective doses. Glioblastoma cells were found to be more sensitive to TQ-induced anti-proliferative effect as compared to normal cells (IMR90 and hTERT-BJ1). Reduction in cell viability was attributed to the induction of apoptosis by TQ in glioblastoma cells as evidenced by higher percentage of cells with Annexin V staining and more cells in sub-G1 phase of the cell cycle following treatment.

Majority of the cancer chemotherapy drugs function as DNA damaging agents that subsequently trigger cell death. In this study, we show that TQ induces DNA damage and cell death in glioblastoma cells. When DNA damages are irreparable, the cells undergo apoptosis. Induction of DNA damage in normal cells led to p53 mediated p21 triggered growth inhibitory effects which potentially allow repair. However, TQ treatment blocked glioblastoma cell growth and induced cell killing by apoptosis. We believe that the apoptotic effect of TQ was modulated by increased expression of Bax and cytochrome *c* proteins. Apoptosis *via* mitochondrial pathway involves the release of the inter-membrane mitochondrial proteins, cytochrome *c* into cytosol and subsequently triggering a cascade of proteolytic events [25]. Based on our findings, we speculate that TQ induces apoptosis in glioblastoma cells proficient in DNA-PKcs by the above-mentioned mechanism, which was evidenced by an increase in the cytosolic cytochrome *c* level. Studies have shown that TQ induces apoptosis by both p53-dependent [24] and p53-independent pathways [26]. Our data agrees with the latter wherein cell death triggered by TQ in glioblastoma was p53 independent as shown by the minimal change in expression of p53.

Tumour cells develop resistance to chemotherapy or radiotherapy over time via genetic alteration resulting in less effective treatment. DNA-PKcs is essential for DNA double strand breaks repair and maintenance of genomic integrity [27]. Studies have shown that DNA double strand break repair in cells deficient in DNA-PKcs usually results in high frequencies of mis-repair, chromosome aberrations and complex changes [12]. In our study, we show that glioblastoma cells with different DNA-PKcs status respond differently to TQ exposure. DNA-PKcs deficient glioblastoma cells displayed significant cell cycle arrest at G_2_/M phase in addition to cell death. Significant level of TQ induced DNA damage was also observed in these cells at lower dose of TQ. This observation correlates with earlier findings that only DNA-PKcs deficient glioblastoma cells showed G_2_/M accumulation upon treatment with DNA double strand break inducing agents [28]. Moreover, the frequency of binucleated cells with micronuclei, indicative of chromosome instability, was higher in DNA-PKcs deficient glioblastoma cells as compared to DNA-PKcs proficient glioblastoma cells (data not shown). This points out that although DNA-PKcs deficient glioblastoma cells were less sensitive to TQ induced cytotoxicity as compared to DNA-PKcs proficient glioblastoma cells, greater extent of genomic instability could be detected in DNA-PKcs deficient glioblastoma which might be due to the inefficient non-homologous end joining DNA repair processes. The increased cell death shown in DNA-PKcs proficient glioblastoma cells supports the view that TQ targets DNA-PKcs proficient glioblastoma cells and that DNA-PKcs activation may play a role in apoptosis. This finding corroborates an earlier study, which showed a greater resistance of DNA-PKcs deficient glioblastoma cells to cell death as compared to DNA-PKcs proficient glioblastoma [29].

Interestingly, telomerase positive human foreskin fibroblast cells (hTERT-BJ1) were also more sensitive to growth inhibitory effect of TQ as compared to normal lung fibroblasts (IMR90). Telomerase activity was evaluated following TQ treatment to hTERT-BJ1 cells to determine whether increased sensitivity was due to telomerase inhibition. Telomerase is essential in regulating telomere length in immortal cells as well as maintaining the proliferative capacity of cells [8], [30], [31], [32]. Telomerase may play a central role in cellular resistance to apoptosis of cancer cells and inhibition of telomerase may induce apoptosis [33], [34]. Telomere dysfunction, whether due to replicative shortening or experimental disruption of telomeric DNA-protein complex structure, leads to rapid DNA damage response which can ultimately induce senescence and/or apoptosis [35]. Attempts are being made to exploit this aspect in cancer therapy. In our study, for the first time, we found that TQ significantly reduces telomerase activity and induces telomere attrition. A significant reduction in telomerase activity and expression of hTERT protein was observed in hTERT-BJ1 and M059K cells and not M059J cells following TQ treatment. Among the cancer cells, we observed that the level of telomerase activity decreased significantly only in DNA-PKcs proficient glioblastoma cells with respect to its controls. Similar results were obtained with regards to TQ mediated telomere attrition which can be attributed to the selective inhibition of telomerase activity in DNA-PKcs proficient cells.

The differential response of M059J and M059K gliobloastoma cells to TQ treatment prompted us to characterise these cells to determine whether defective DNA-PKcs status has resulted in any specific changes in the basal genetic traits. Earlier, we had shown that DNA-PKcs is critical for telomere capping [36], [37] apart from its function in responding to DNA damage. DNA-PKcs deficient mouse embryonic fibroblasts displayed higher telomere fusions and chromosome instability [36]. Considering the role of DNA-PKcs at telomeres, we went on to validate our observation on the role of DNA-PKcs in TQ mediated telomere attrition. Subtle differences in the karyotypes of M059K and M059J cells were identified and these cells harbour several recurrent complex chromosomal rearrangements. In order to further the understanding if the above findings, gene expression profiling was done in these cell lines. A total 587 genes (∼2% of the total genes profiled) were differentially expressed based on the set analysis criteria. However, more work needs to be done to validate the functional relevance of these genes and whether they are dependent on the status of DNA-PKcs. All in all, there are differences in the chromosome rearrangements and gene expression profiles of two glioblastoma cell types used in the study but the gross genetic changes might be minimal in the two glioblastoma cell types used in the study. Telomere-mediated chromosome instability was commonly detected in cells deficient in DNA-PKcs [36], [38], [39], [40]. Therefore, we believe that defective DNA-PKcs might be the cause of differential response to TQ in our study.

In order to validate and substantiate our data on DNA-PKcs deficient M059J cells, we used NU7026 to inhibit the kinase activity of DNA-PKcs in M059K cells to study the telomere attrition following TQ treatment. Pre-treatment with NU7026 in M059K cells produced a similar response to that of M059J cells for TQ exposure. Therefore, it is clear that TQ mediated effects on telomerase and telomere length were significantly reduced in the absence of DNA-PKcs achieved either by using a DNA-PKcs deficient cell type or by functional inhibition of its kinase activity in DNA-PKcs proficient cells. We therefore, suggest the DNA-PKcs activity is rather relevant to mediate the above observed TQ effects. It was recently reported that the absence of DNA-PKcs increases the spontaneous telomere attrition rate in telomerase knockout (mTERC^−/−^) mice [41]. Based on our results, we can cautiously speculate that DNA-PKcs activity is relevant to specifically facilitate TQ effects. Further investigation is warranted to find out the factors downstream of DNA-PKcs which are required for external agent induced telomere attrition in human cells. In conclusion, we demonstrate that TQ induces higher induction of apoptosis in glioblastoma cells. Furthermore, we could detect telomerase inhibition, telomere attrition, increased DNA damage and apoptosis by TQ in DNA-PKcs proficient glioblastoma cells as compared to DNA-PKcs deficient glioblastoma cells. In addition, TQ also mediates apoptosis independent of telomerase attrition as observed in DNA-PKcs deficient glioblastoma cells. Thus, this agent can be a potential candidate as chemotherapeutic agent for the treatment of brain cancers. However, detailed studies are required to profile the genome wide effects of TQ to exploit its therapeutic potential more effectively.

## References

[1] Vuorelaa P, Leinonenb M, Saikkuc P, Tammelaa P, Rauhad JP (2004). Natural products in the process of finding new drug candidates.. Curr Med Chem.

[2] Aggarwal BB, Kunnumakkara AB, Harikumar KB, Tharakan ST, Sung B (2008). Potential of spice-derived phytochemicals for cancer prevention.. Planta Med.

[3] Padhye S, Banerjee S, Ahmad A, Mohammad R, Sarkar FH (2008). From here to eternity - the secret of Pharaohs: Therapeutic potential of black cumin seeds and beyond.. Cancer Ther.

[4] Yi T, Cho SG, Yi Z, Pang X, Rodriguez M (2008). Thymoquinone inhibits tumor angiogenesis and tumor growth through suppressing AKT and extracellular signal-regulated kinase signaling pathways.. Mol Cancer Ther.

[5] Shoieb AM, Elgayyar M, Dudrick PS, Bell JL, Tithof PK (2003). In vitro inhibition of growth and induction of apoptosis in cancer cell lines by thymoquinone.. Int J Oncol.

[6] Worthen DR, Ghosheh OA, Crooks PA (1998). The in vitro anti-tumor activity of some crude and purified components of blackseed, Nigella sativa L.. Anticancer Res.

[7] de Lange T, DePinho RA (1999). Unlimited mileage from telomerase?. Science.

[8] Shay JW, Wright WE (2002). Telomerase: a target for cancer therapeutics.. Cancer Cell.

[9] Shay JW, Wright WE (2005). Mechanism-based combination telomerase inhibition therapy.. Cancer Cell.

[10] Falchetti ML, Pallini R, D'Ambrosio E, Pierconti F, Martini M (2000). In situ detection of telomerase catalytic subunit mRNA in glioblastoma multiforme.. Int J Cancer.

[11] Shiraishi S, Tada K, Nakamura H, Makino K, Kochi M (2002). Influence of p53 mutations on prognosis of patients with glioblastoma.. Cancer.

[12] Virsik-Kopp P, Rave-Frank M, Hofman-Huther H, Schmidberger H (2003). Role of DNA-PK in the process of aberration formation as studied in irradiated human glioblastoma cell lines M059K and M059J.. Int J Radiat Biol.

[13] Jeggo PA (1997). DNA-PK: at the cross-roads of biochemistry and genetics.. MutatRes.

[14] Smith GC, Jackson SP (1999). The DNA-dependent protein kinase.. Genes Dev.

[15] Willmore E, de CS, Sunter NJ, Tilby MJ, Jackson GH (2004). A novel DNA-dependent protein kinase inhibitor, NU7026, potentiates the cytotoxicity of topoisomerase II poisons used in the treatment of leukemia.. Blood.

[16] Poonepalli A, Balakrishnan L, Khaw AK, Low GK, Jayapal M (2005). Lack of poly(ADP-ribose) polymerase-1 gene product enhances cellular sensitivity to arsenite.. Cancer Res.

[17] d'Adda di Fagagna F, Hande MP, Tong WM, Lansdorp PM, Wang ZQ (1999). Functions of poly(ADP-ribose) polymerase in controlling telomere length and chromosomal stability.. Nat Genet.

[18] Hande MP, Samper E, Lansdorp P, Blasco MA (1999). Telomere length dynamics and chromosomal instability in cells derived from telomerase null mice.. J Cell Biol.

[19] Hande MP, Azizova TV, Burak LE, Khokhryakov VF, Geard CR (2005). Complex chromosome aberrations persist in individuals many years after occupational exposure to densely ionizing radiation: an mFISH study.. Genes Chromosomes Cancer.

[20] Hande MP, Azizova TV, Geard CR, Burak LE, Mitchell CR (2003). Past exposure to densely ionizing radiation leaves a unique permanent signature in the genome.. Am J Hum Genet.

[21] lalunis-Turner MJ, Barron GM, Day RS, Dobler KD, Mirzayans R (1993). Isolation of two cell lines from a human malignant glioma specimen differing in sensitivity to radiation and chemotherapeutic drugs.. Radiat Res.

[22] Lees-Miller SP, Godbout R, Chan DW, Weinfeld M, Day RS (1995). Absence of p350 subunit of DNA-activated protein kinase from a radiosensitive human cell line.. Science.

[23] Sfeir A, Kosiyatrakul ST, Hockemeyer D, MacRae SL, Karlseder J (2009). Mammalian telomeres resemble fragile sites and require TRF1 for efficient replication.. Cell.

[24] Gali-Muhtasib H, ab-Assaf M, Boltze C, Al-Hmaira J, Hartig R (2004). Thymoquinone extracted from black seed triggers apoptotic cell death in human colorectal cancer cells via a p53-dependent mechanism.. Int J Oncol.

[25] Li K, Li Y, Shelton JM, Richardson JA, Spencer E (2000). Cytochrome c deficiency causes embryonic lethality and attenuates stress-induced apoptosis.. Cell.

[26] El-Mahdy MA, Zhu Q, Wang QE, Wani G, Wani AA (2005). Thymoquinone induces apoptosis through activation of caspase-8 and mitochondrial events in p53-null myeloblastic leukemia HL-60 cells.. Int J Cancer.

[27] Kurimasa A, Kumano S, Boubnov NV, Story MD, Tung CS (1999). Requirement for the kinase activity of human DNA-dependent protein kinase catalytic subunit in DNA strand break rejoining.. Mol Cell Biol.

[28] Holgersson A, Heiden T, Castro J, Edgren MR, Lewensohn R (2005). Different G2/M accumulation in M059J and M059K cells after exposure to DNA double-strand break-inducing agents.. Int J Radiat Oncol Biol Phys.

[29] Chen GG, Sin FL, Leung BC, Ng HK, Poon WS (2005). Glioblastoma cells deficient in DNA-dependent protein kinase are resistant to cell death.. J Cell Physiol.

[30] Blackburn EH (2001). Switching and signaling at the telomere.. Cell.

[31] Blackburn EH, Greider CW, Szostak JW (2006). Telomeres and telomerase: the path from maize, Tetrahymena and yeast to human cancer and aging.. Nat Med.

[32] Shay JW, Bacchetti S (1997). A survey of telomerase activity in human cancer.. Eur J Cancer.

[33] Herbert B, Pitts AE, Baker SI, Hamilton SE, Wright WE (1999). Inhibition of human telomerase in immortal human cells leads to progressive telomere shortening and cell death.. Proc Natl Acad Sci USA.

[34] Kondo Y, Kondo S, Tanaka Y, Haqqi T, Barna BP (1998). Inhibition of telomerase increases the susceptibility of human malignant glioblastoma cells to cisplatin-induced apoptosis.. Oncogene.

[35] Herbig U, Jobling WA, Chen BP, Chen DJ, Sedivy JM (2004). Telomere shortening triggers senescence of human cells through a pathway involving ATM, p53, and p21(CIP1), but not p16(INK4a).. Mol Cell.

[36] Gilley D, Tanaka H, Hande MP, Kurimasa A, Li GC (2001). DNA-PKcs is critical for telomere capping.. Proc Natl Acad Sci U S A.

[37] Hande P, Slijepcevic P, Silver A, Bouffler S, van Buul P (1999). Elongated telomeres in scid mice.. Genomics.

[38] Bailey SM, Brenneman MA, Halbrook J, Nickoloff JA, Ullrich RL (2004). The kinase activity of DNA-PK is required to protect mammalian telomeres.. DNA Repair (Amst).

[39] Hande MP (2004). DNA repair factors and telomere-chromosome integrity in mammalian cells.. Cytogenet Genome Res.

[40] Williams ES, Klingler R, Ponnaiya B, Hardt T, Schrock E (2009). Telomere dysfunction and DNA-PKcs deficiency: characterization and consequence.. Cancer Res.

[41] Espejel S, Franco S, Sgura A, Gae D, Bailey SM (2002). Functional interaction between DNA-PKcs and telomerase in telomere length maintenance.. EMBO J.

